# Functional characterization of a serine-threonine protein kinase from *Bambusa balcooa* that implicates in cellulose overproduction and superior quality fiber formation

**DOI:** 10.1186/1471-2229-13-128

**Published:** 2013-09-10

**Authors:** Jayadri Sekhar Ghosh, Shubho Chaudhuri, Nrisingha Dey, Amita Pal

**Affiliations:** 1 Bose Institute, P1/12 CIT Scheme VIIM, Kolkata, India; 2 Institute of Life Sciences, Chandrasekharpur, 751023 Bhubaneswar, Odhisa, India; 3 Present Address: Department of Agronomy, Iowa State University, 50011-1010 Ames, Iowa, USA

**Keywords:** *Bambusa balcooa*, Fiber, Receptor like cytoplasmic kinase, SCAR marker, Serine-threonine, Kinase, Leucine rich repeat domain, Kinase domain

## Abstract

**Background:**

Molecular markers allow rapid identification of biologically important germplasm/s having desired character. Previously we have reported a genotype specific molecular marker, Balco_1128_ [GenBank ID EU258678] of *Bambusa balcooa* containing an ORF (375 bp) having high similarity with receptor like cytoplasmic kinase of *Arabidopsis* and *Oryza*. Balco_1128_ was found to be associated only with bamboo genotypes endowed with high cellulose and low lignin contents of fibers. Under the above backdrop, it was necessitated to characterize this genetic marker for better understanding of its biological significance in context of superior quality fiber development.

**Results:**

The full length cDNA (3342 bp) of BbKst, a serine-threonine protein kinase was isolated from *B. balcooa* comprising of six LRR domains at the N-terminal end and a kinase domain at the C-terminal end. Bacteria-expressed BbKst-kinase domain (3339 bp long) showed Mg^2+^ dependent kinase activity at pH 7.0, 28°C. Bioinformatics study followed by phospho-amino analysis further confirmed that BbKst-kinase belongs to the serine/threonine protein kinase family. Transcript analysis of the *BbKst* gene following RNA slot blot hybridization and qPCR revealed higher expression of *BbKst* during initiation and elongation stages of fiber development. Tissue specific expression studies showed much higher expression of *BbKst* transcript in stems and internodes of *B. balcooa* than in leaves and rhizomes. Southern analysis revealed single copy insertion of *BbKst* in most of the *Agrobacterium* mediated transgenic tobacco plants. Real-time PCR detected 150-200 fold enhanced expression of *BbKst* in different T_1_ tobacco lines than that of the vector transformed plants. Heterologous expression of *BbKst* under control of 35S promoter in transgenic tobacco showed high cellulose deposition in the xylem fibers. Number of xylary fibers was higher in transgenic T_0_ and T_1_ plants than that of empty-vector transformed tobacco plants offering enhanced mechanical strength to the transgenic plants, which was also substantiated by their strong upright phenotypes, significantly higher cellulose contents, flexibility coefficient, slenderness ratio, and lower Runkel ratio of the fibers.

**Conclusions:**

This finding clearly demonstrated that *BbKst* gene (GenBank ID JQ432560) encodes a serine/threonine protein kinase. *BbKst* induced higher cellulose deposition/synthesis in transgenic tobacco plants, an important attribute of fiber quality bestowing additional strength to the plant.

## Background

*Bambusa balcooa* is an abundant tropical species and recognized by Food and Agriculture Organization of the United Nations as a priority bamboo species amongst eighteen other bamboo species distributed globally [1]. It can quickly restore forest flora and the pre-existing environmental milieu due to its faster growth rate. *B. balcooa* has better physical and mechanical properties and is used as raw material for paper and pulp industries. In India alone, about 2.2 million tonnes of bamboo are used in paper industries [2]. The superior commercially useful traits of *B. balcooa* demanded isolation and cloning of gene contributing towards superior fiber quality production.

Bioprospecting of genes from any non-model tree species for biotechnological applications is mainly focused on the development of molecular markers. Molecular markers allow rapid identification of the gene or genes of interest from genomic DNA. In our laboratory, we had developed a genotype specific SCAR (sequence characterized amplified region) marker, Balco1128 (GenBank ID EU258678) [3] with 375 bp long ORF, having high similarities with a protein of *Arabidopsis thaliana* (GenBank ID gi15219675) and a putative protein kinase of *Oryza sativa* cv. Japonica (GenBank ID gi50918597). The marker, Balco_1128_ was found to be associated with high cellulose containing fibers of *B. balcooa* genotypes [3]. Subsequently the marker, Balco_1128_ was employed to screen elite genotypes of *B. balcooa* for the efficient commercial utilization of the available superior natural germplasm resources.

There are limited reports on fiber related kinases. It was shown that a receptor-like kinase (RLK) gene of cotton (GhRLK1) participates in cotton fiber development and fiber cell wall synthesis [4]. Subsequently, one kinase EST (GenBank ID GW820201) [5] has been identified from bamboo which has higher expression during fiber cell initiation and elongation. Protein kinases represent a biologically significant group of plant proteins as they are implicated in several cellular functions including cell proliferation, differentiation and cell death mediating cell signaling pathways. Of these, the receptor-like protein kinases (RLKs) form one of the largest and most diverse super-families of plant proteins [6]. RLKs have diverse biological roles, such as plant growth and development, hormonal response, cell differentiation, self-incompatibility, stress response and pathogen recognition [7,8]. The Receptor Like Cytoplasmic Kinase (RLCK) gene family codes for proteins lacking the transmembrane domain found in the typical RLKs [6]. The annotated Arabidopsis genome shows around 600 RLK-coding genes out of which around 200 have no extracellular domain; suggesting involvement of RLCK family in growth and developmental processes of plants [6].

Plant RLKs are classified into several subclasses, the largest of which is the sub-family of LRR receptor-like kinases [9]. LRR-RLKs were studied extensively and it was found that this group of proteins plays important roles in diverse processes of plant development [10,11]. Under the above backdrop, in the present study we made an attempt to isolate full length sequence of this putative leucine rich kinase gene employing Balco_1128_ sequence information, RNA ligase mediated (RLM)- Rapid Amplification of cDNA Ends (RACE) and genome walking.

*In silico* sequence analysis confirms the presence of kinase and LRR domains within BbKst. The protein encoded by the kinase domain of *BbKst* gene was expressed in *Escherichia coli*, purified to determine the nature of this putative protein kinase. Biochemical analyses of the protein kinase were conducted and the nature and kinase activity of the protein has been deciphered.

Subsequently, transgenic tobacco plants were raised and molecular analyses were conducted. Transcripts of *BbKst* were quantified in GUS positive transgenic T_1_ tobacco plants using qPCR. Cellulose contents of the fibers isolated from transgenics and that of empty-vector transformed control tobacco plants were analysed. Physical parameters of fibers including flexibility coefficient, slenderness ratio and Runkel ratios of transgenics were compared with that of vector-transformed tobacco plants to reveal whether *BbKst* confers any additive value in fiber quality as noted in superior genotypes of *B. balcooa*. Confocal laser scanning microscopy (CLSM) with cellulose specific staining of stem cross sections of transgenics and that of vector-transformed plants was undertaken at the T_0_ and T_1_ generations to demonstrate *in situ* distribution of cellulose.

The present finding demonstrated that *BbKst* (GenBank ID JQ432560) encodes a serine/threonine protein kinase that enhances higher cellulose deposition/synthesis, an important attribute that confers superior fiber quality. This was envisaged in our previous study [3], wherein we showed that the presence of Balco_1128_, which is a part of *BbKst* gene, is associated only with high cellulose containing *B. balcooa* genotypes. The present study also has shown that *BbKst* induced formation of expanded xylary zone and enhanced cellulose synthesis in the transgenic tobacco plants bestowing additional mechanical strength. Thus, this finding revealed why the marker ‘Balco_1128_’ is genotypically specific and present only in high cellulose containing superior-fiber-yielding genotypes as reported by Pal et al. [3].

## Results

### Analysis of the *BbKst* gene architecture

**Figure 1:**
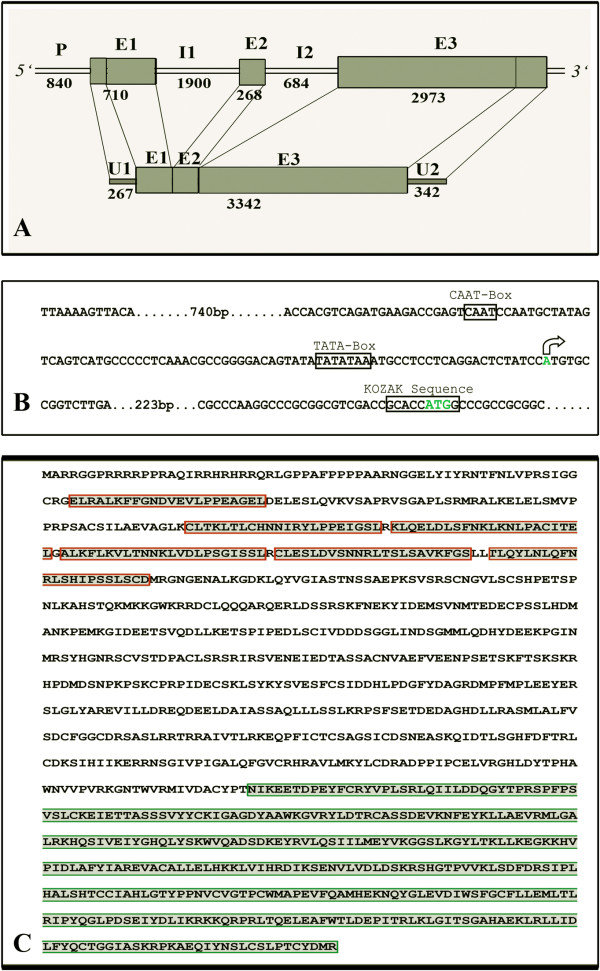
**Schematic diagram of *BbKst *gene structure. A**. Genomic organization of total *BbKst* sequence. P = promoter; E = exon; I = intron, U = UTR region; sequence length of all these regions are mentioned in bp at the respective positions. **B**. Predicted organizational pattern of *BbKst* promoter, major sites mentioned within the black box. Position of the transcription start site is shown by an arrow. **C**. Predicted amino acid residues of BbKst protein, red lined boxes representing LRR repeats and the green box at the C terminal end represents kinase domain.

**Figure 2:**
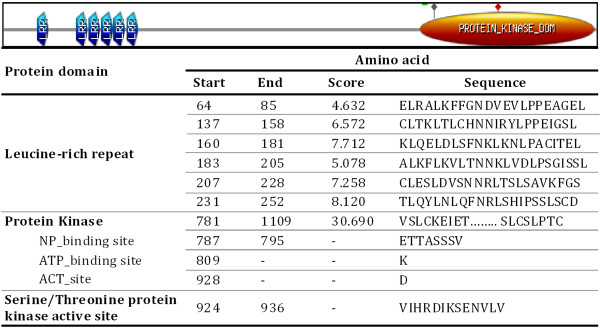
**Prosite scan of the BbKst.** Start and end positions of the amino acid residues and peptide sequences of various protein domains are shown.

The full length *BbKst* gene (7375 bp) was isolated using RACE and genome walking. A representative gel documenting RACE products and genome walked PCR products are shown in Additional file 1: Figure S1 Ai, Aii. The *BbKst* sequence (GenBank ID JQ432560) was subjected to *in silico* analysis, which showed a 3342 bp long coding region with three exons of 443 (E1), 268 (E2) and 2631 (E3) bp sizes, separated by two introns (1900 and 684 bp) as shown in Figure 1. The putative start (ATG) and stop (TGA) codons were positioned at 265 and 7033 bp, from the transcription start site, respectively, and a poly-adenylation motif AATAAA was found at the end of the 3′ UTR sequence. A schematic diagram of the *BbKst* sequence organization is shown in Figure 1A. A putative promoter sequence, 840 bp in length, was analysed by using PlantCare (http://bioinformatics.psb.ugent.be/webtools/plantcare/html/) and PlantPAN (http://plantpan.mbc.nctu.edu.tw/gene_group/index.php), and contains several specific structural elements including a putative TATA box (-34 to -29), CAAT box (–81 to –78), Kozak sequence (GCACCATGG) at the positions -5 to +3; with a cluster of important plant specific key regulatory cis-elements (Figure 1B; Additional file 2: Table S1). DNAMAN based analysis of 3951 bp long cDNA sequence revealed that it contains 267 and 342 bp long UTR regions at the 5′ and 3′ ends, respectively. Further analysis of *BbKst* gene using NCBI-CDS software has clearly shown that the predicted BbKst protein has distinct PKC domain and LRR regions. Exact orientation of the kinase domain and LRR repeats were recognized with a Pfam search (Figure 1C). Positions of six LRR repeat regions, ATP binding site, active sites and nucleotide phosphate-binding region were determined by PROSITE and results are depicted in Figure 2. Domain analysis also indicates that BbKst protein has no extracellular domain indicating that it belongs to RLCK subfamily.

### Analysis of biochemical properties of BbKst-kinase

**Figure 3:**
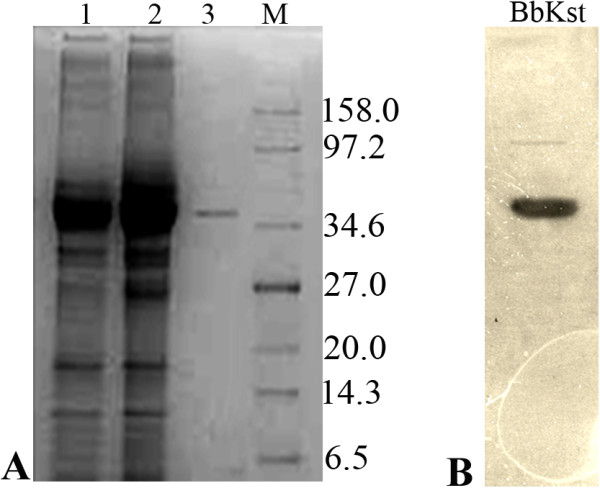
**BbKst protein after purification. A**. BbKst-kinase expression in bacterial cells, Lane 1. Total protein in soluble fraction, Lane 2. Total protein in insoluble fraction and Lane 3. Purified protein isolated from the soluble fraction. M = molecular weight marker. **B**. Western blot of the purified protein against anti-his antibody.

The kinase domain of the BbKst protein was expressed in *E. coli* BL21 cells as described in the methods section. Expression of BbKst-kinase was found in both soluble and insoluble fractions of total cell lysate (Figure 3A). Subsequently, the purified protein was confirmed by western blot (Figure 3B).

**Figure 4:**
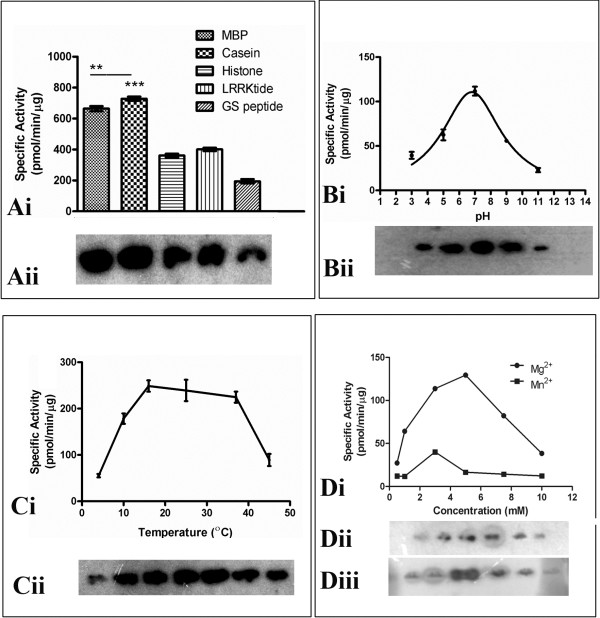
**Biochemical properties of BbKst-kinase. Ai**. Specific activity of BbKst on different substrates. The significant differences between specific activities with different substrates were determined by One-way ANOVA, ** and *** represents p < 0.001 and 0.0001, respectively. Each experiment was performed in triplicates, and the average data presented. **Aii**. Autoradiogram image of the substrate phosphorylation. **B**. pH dependent curve. Enzyme assay was performed at pH 3 to 11. The graph was fitted following Lorentzian equation. Error bars indicate standard deviation between three replicates **(Bi)**. Respective autoradiogram of the substrate phosphorylation at different pH **(Bii)**. **C**. Specific activity of BbKst-Kinase at different temperature from 4 to 45ºC using casein as substrate. Error bars indicate standard deviation between three replicates **(Ci)**. Respective autoradiogram of the substrate phosphorylation at different pH **(Cii)**. **Di**. Casein phosphorylation activity of BbKst-kinase in presence of different concentrations of Mg^2+^ or Mn^2+^ was determined in presence of 0.5, 1, 2.5, 5, 7.5 and 10 mM of Mn ^2+^ (source MnCl_2_) or Mg^2+^ (source MgCl_2_). Respective autoradiograms of phosphorylation in presemce of Mn ^2+^**(Dii)** and Mg^2+^**(Diii)**.

BbKst-kinase phosphorylates all the substrates tested in the present study viz., casein, myelin basic protein (MBP), histone (H1), ‘LRRKtide’ and ‘GS’ peptide (Figure 4Ai). The respective phosphorylated products were electrophoresed and the auto-radiographed image is shown in Figure 4Aii. Among these substrates, the protein showed higher specific activity with casein and MBP than others (Figure 4Ai). Therefore, we used casein as a substrate in the subsequent experiments to study the biochemical properties of BbKst-kinase. Casein phosphorylation was determined over a pH range from 3 to 11 and subsequent specific activity showed a bell shaped pattern within the above said pH range (Figure 4Bi). The BbKst kinase showed highest activity at pH 7.0 when casein was used as the *in vitro* substrate (Figure 4Bii). The effect of temperature on casein phosphorylation by the BbKst-kinase was also determined. The substrate phosphorylation activity of the BbKst-kinase showed a gradual increase in activity from 4 to 15°C, followed by an optimum stationary stage up to 40°C and then gradually decreased with increasing temperature (Figure 4Ci, Cii).

**Figure 5:**
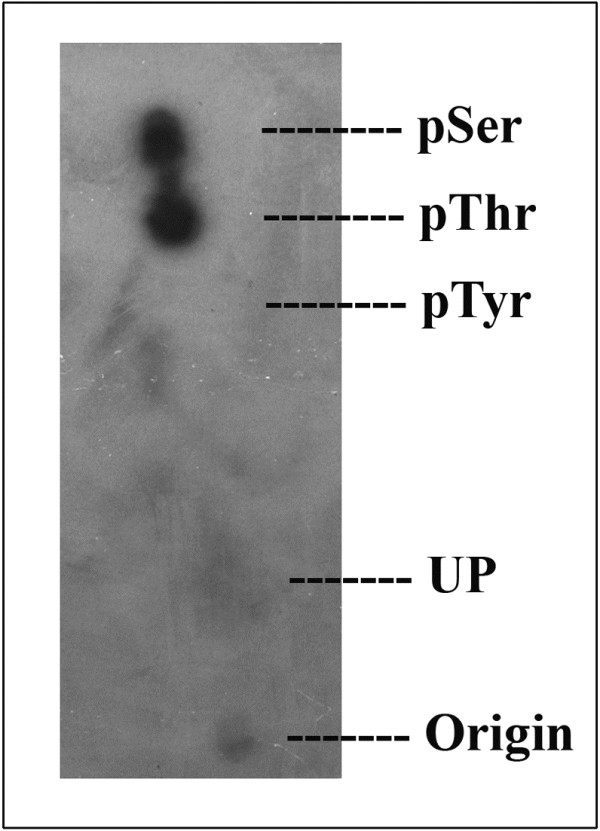
**Phospho-amino acid analysis of BbKst-kinase by thin-layer chromatography. **^32^P-labeled protein, after *in vitro* kinase reaction, was acid hydrolyzed. Hydrolyzed labelled amino acids were spotted along with three authentic markers of pSer, pThr, and pTyr amino acids on cellulose thin-layer chromatography plates and separated at high voltage. Marker phosphoamino acids were detected by ninhydrin staining; whereas, labeled phosphoamino acids were detected by autoradiography. UP represents unhydrolyzed protein.

BbKst-kinase showed highest activity in presence of Mg^2+^. It was found that casein phosphorylation by BbKst-kinase increased along with increased concentration of Mg^2+^ from 0.5 or 5.0 mM, while, in the case of Mn^2+^ a weak phosphorylation of casein was noted only at 2.5 mM (Figure 4D) indicating that BbKst-kinase prefer Mg^2+^ over Mn^2+^ for its enzyme activity. Phosphoamino acid analysis of autophosphorylated BbKst revealed that BbKst phosphorylated only serine and threonine residues but not tyrosine (Figure 5).

### Enzyme kinetics of BbKst-kinase

**Figure 6:**
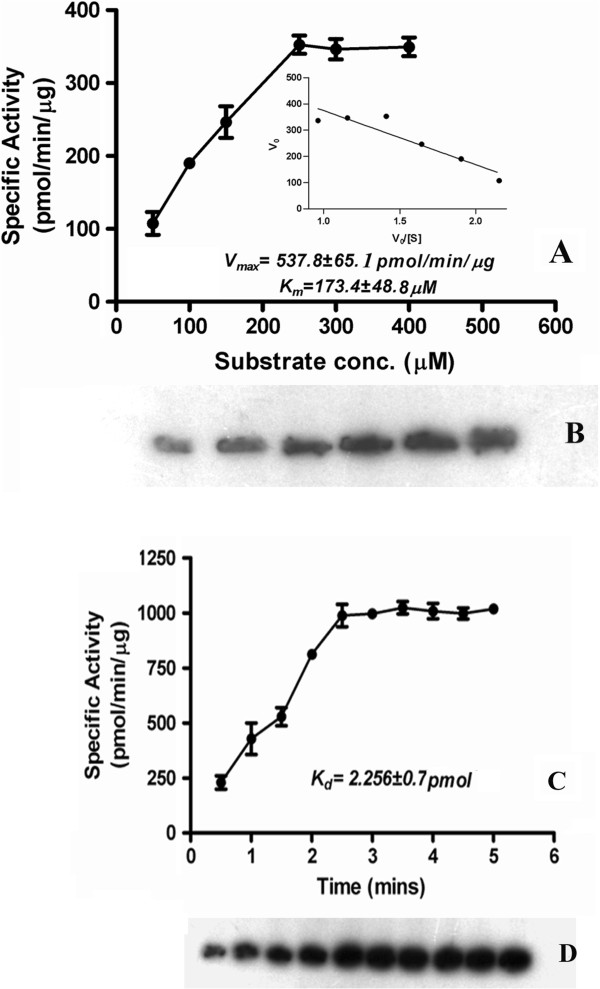
**Saturation curve of BbKst activity at different concentrations of casein. A**. Data represents average of three independent assays of specific activity of the enzyme in presence of different casein concentrations. Eadie-Hofstee plot of the average values for each data set is shown in the inset. Error bars indicate ± SD (n = 3**)**. **B**. Autoradiogram at different substrate concentrations. **C**. Time-dependent activation of BbKst. Average data of three independent assays presented as saturation curves with specific activity versus casein concentration as indicated. Error bars indicate ± SD (n = 3). Dissociation constant for Kd of both the reactions are shown within the graphs. **D**. Autoradiogram of casein phosphorylation at different time intervals.

BbKst exhibited Michaelis-Menten kinetics with respect to casein as substrate. The *Vmax* and *K*m value of BbKst-kinase for casein determined from Eadie-Hofstee plots of V_0_ versus V_0_/[S] were 537.8 ± 65.08 pmol/min/μg and 173.4 ± 48.78 μM, respectively (Figure 6A). The *Kcat* (ratio of *V*max to *K*m) was determined and the value was found to be ~ 3.01 min-1. Casein-phosphorylation of BbKst-kinase showed a steady increase along with time upto 3 min, which remain steady upto 5 min. The dissociation constant (*Kd*) for substrate phosphorylation was computed as 2.3 ± 0.7 pmol (Figure 6B).

### Effect of protein kinase inhibitors on specific activity of BbKst

**Figure 7:**
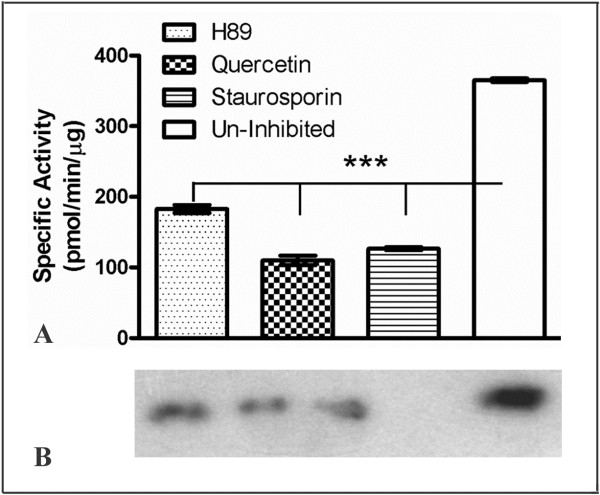
**Effects of different inhibitors on specific activity of BbKst-kinase. A**. Error bars indicate ± SD (n = 3). The significant difference between IC 50 values of different inhibitors was determined by One-way ANOVA, *** represents p < 0.0001. **B**. Autoradiogram of the corresponding gel showing intensities of different inhibitor in respective order.

None of the three inhibitors (quercetin, H-89 and staurosporin) tested could inhibit completely the activity of BbKst-kinase (Figure 7A). Of these three, quercetin showed significantly higher inhibition than H-89 and staurosporine. The IC50 value for quercetin was estimated at 7 nM. ANOVA results showed there is significant difference (P < 0.0001) in IC50 values of these three kinase inhibitors. The products were electrophoresed in SDS-PAGE and a corresponding autoradiogram of the gel is presented in Figure 7B.

### Spatial expression of *BbKst* transcripts in *B. Balcooa*

**Figure 8:**
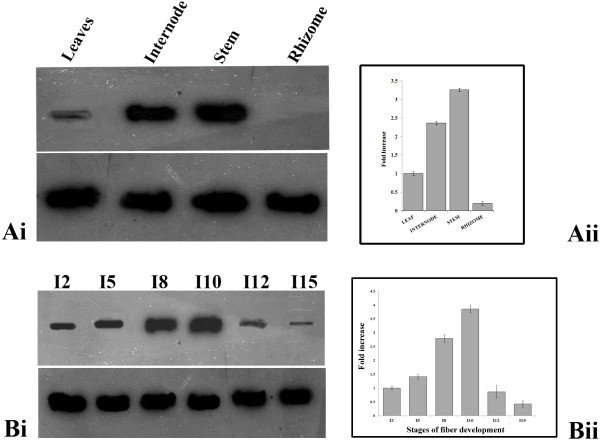
**Expression of *BbKst *transcripts in *B. balcooa*. Ai**. RNA slot blots showing hybridization of a gene specific probe with total RNA from different parts of *B. balcooa*; in top panel lanes were loaded with 20 μg of total RNA from different organs. Bottom panel showed transcript accumulation of GAPDH, similar pattern signifies equal loading. **Aii**. Side panel showed the qPCR analysis of transcript accumulation in different organs. **Bi**. RNA slot blots showing hybridization of a gene specific probe with total RNA from different stages of developing internodes of *B. balcooa*; in top panel lanes were loaded with 20 μg of total RNA from different organs; **I** signifies the internode number. Bottom panel showed transcript accumulation of GAPDH, similar pattern signifies equal loading. **Bii**. Side panel showed the qPCR analysis of transcript accumulation at different developmental stages of internodes.

Differential expression of the *BbKst* transcripts in the stem, internode, rhizome and leaf of *B. balcooa* was noted and confirmed through three independent RNA slot blot hybridization as described in the method section. Results obtained were found to be highly reproducible. The strongest expression of the *BbKst* transcripts was found in mature stem while weaker expression was observed in rhizome. Leaf and internodal tissue showed moderate expression of *BbKst* transcript (Figure 8Ai). All the above findings were further confirmed by qPCR analysis, wherein a weaker expression of the transcript in the rhizome was detected (Figure 8Aii).

### Differential expression of *BbKst* transcript at different fiber developmental stages of *B. balcooa*

Expression analyses of the *BbKst* transcript at different internodal tissue (2^nd^, 5^th^, 8^th^, 10^th^, 12^th^, and 15^th^) revealed highest expression of *BbKst* in the 10^th^ internode followed by 8^th^ (Figure 8Bi). Expression level gradually decreased with fiber maturity and lowest expression level was noted in the 15^th^ internode. Results obtained were consistent in three independent experiments. The expression level of the *BbKst* was found to be 2.8 fold higher during fiber elongation (8^th^ internode) and 3.85 fold higher during fiber maturation (10^th^ internode) than in the fiber initiation stage. All the above findings were further confirmed by qPCR analysis (Figure 8Bii).

### Analysis of transient expressions of the *BbKst* in plant cell

**Figure 9:**
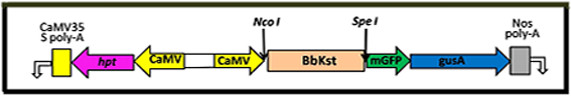
**Construct map of JS1304.** Full length cDNA was inserted in between NcoI and SpeI of the vector pCAMBIA 1304 containing GUS and GFP as selection marker.

**Figure 10:**
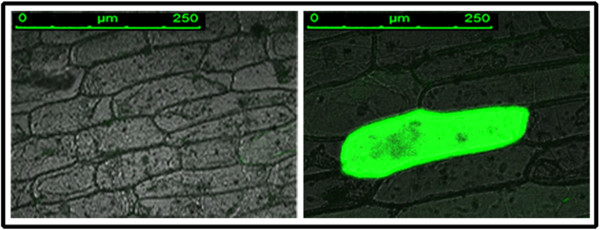
**Transient expression of the BbKst in onion peel.** GFP expression after 24 hr of bombardment in onion cells. Non-transformed onion cells (left panel) and green fluorescing JS1304-transformed onion cell (right panel).

Transient expression of a BbKst-GFP fusion was analysed using onion peels bombarded with plant expression vector pCambia 1304 carrying total cDNA sequence of *BbKst*, designated as Js1304 (Figure 9). Onion epidermal cells exhibited expression of the GFP-fusion protein in the cytoplasmic region of the Js1304 transformed onion cell after 24 hr (Figure 10). Expression was not found in the non-transformed tissue (Figure 10). This observation indicates constitutive expression of GFP reporter in the cytoplasm.

### Development of transgenic tobacco plants with *BbKst* gene

**Figure 11:**
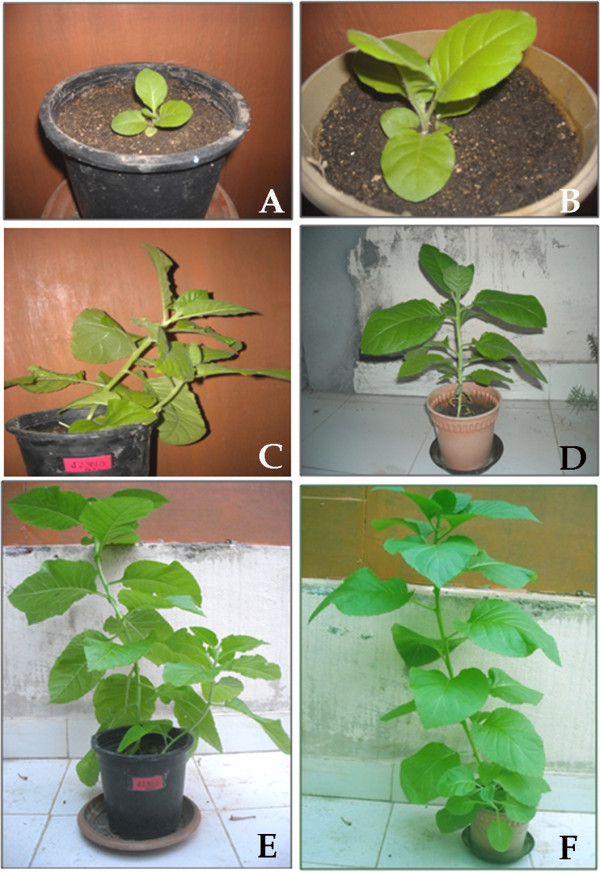
**Phenotypes of transgenic tobacco plants.** Left panel **(A**, **C**, **E)** showing the vector-transformed tobacco plants and right panel **(B**, **D**, **F)** showing transgenic *BbKst* plants. Weak stem is prominent in vector-transformed plants **(C**, **E)** than that of transgenic plants **(D**, **F)** with strong and erect stems.

Tobacco leaf discs were transformed with pCambia 1304 carrying Js1304 and transformants were selected under hygromycin containing medium. Putative transgenic plants were then transferred to soil and T_0_ plants took nearly 6 months to produce flowers and seeds. The comparative phenotypic features of empty-vector transformed and transgenic plants are shown in Figure 11A-F. It was noted that empty vector-transformed plants were gradually stooping from one month onwards after soil transfer (Figure 11C), but the transgenic plants were strong and upright (Figure 11D, F). The stem of vector-transformed plants were comparatively weak than the transgenic plants containing *BbKst* (compare Figure 11E with Figure 11F). Similar observations were also noted in case of T_1_ transgenic tobacco plants (Additional file 3: Figure S2 A-F).

### Selection of GUS positive plants

GUS expression was studied in thirty day old transgenic tobacco seedlings (T_1_) in presence of X-gluc as stated in Method section. Prominent blue colouration was observed in transgenic seedlings (Additional file 4: Figure S3 A-C). Blue colour did not appear in empty vector-transformed seedling (Additional file 4: Figure S3 D).

### Copy number determination of *BbKst*

In most of the T_1_ events the transgene was found to be stably integrated into the genome as a single copy, confirming perfect transgene transmission in the next generation (Additional file 5: Figure S4).The GUS expressive and single copy *BbKst* integrated lines were selected for further study.

### Heterologous expression of *BbKst* in transgenic tobacco

**Figure 12:**
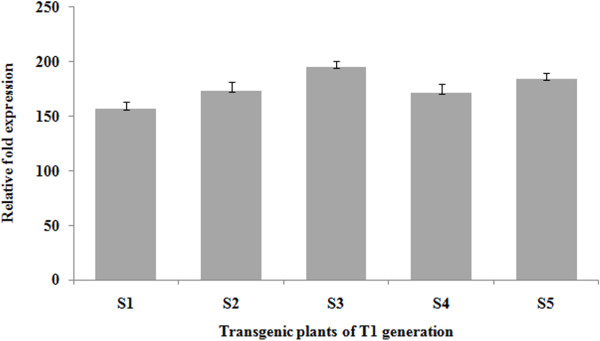
**qPCR analyses of *BbKst *transcripts in transgenic tobacco.** Relative expression level of *BbKst* in T_1_ tobacco lines considering expression in empty vector transformed plant as 1. The data was normalized with reference to 18S rRNA as an internal control. The standard deviation was calculated from three replicates of each sample.

qPCR detected the expression level of *BbKst* in different T_1_ progeny lines. Different transgenic lines exhibited 150-200 fold enhanced expression level than that of the empty vector control tobacco plants. Among five lines of transgenic *BbKst* plants, line 3 on an average showed maximum expression followed by line 4 and 2 (Figure 12).

### Physical and anatomical characteristics of fibers of transgenic plants

**Figure 13:**
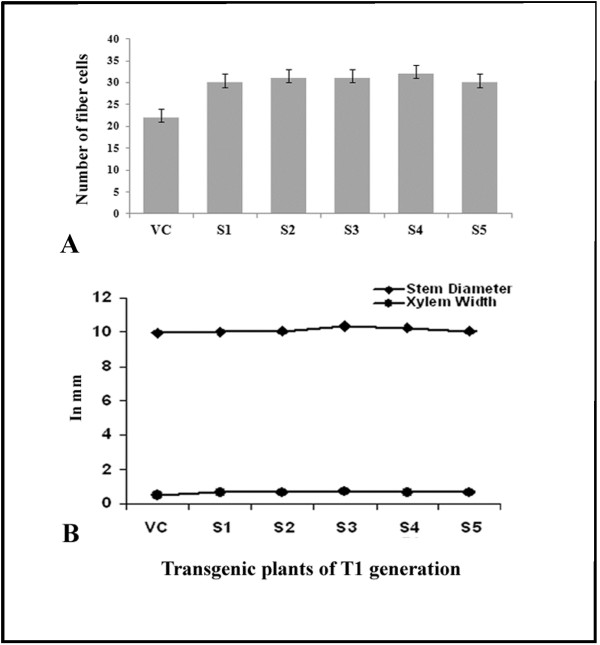
**Stem diameters of 5 transgenic and vector-transformed plants. A**: Bar diagram depicting mean stem diameter of internodal regions of independent transgenic plants and vector-transformed plant. **B**: Width of xylem zone and corresponding stem width of five independent transgenic events at the T_1_ generation. In all cases empty-vector transformed plants were used as control.

Stem diameters of five transgenic plants (S1 to S5) expressing *BbKst* gene and empty vector-transformed tobacco plants (control) were measured and data are represented in Figure 13A. Stem thickness of independent transgenic plants varied from 1.0 cm to 1.04 cm with an average diameter of 1.0 cm. No significant increase in stem diameter of transgenic plants expressing *BbKst* gene over vector-transformed plants was noted. A comparative account between xylem width and stem width of five independent transgenic events at the T_1_ generation is presented in Figure 13B.

The standardized maceration technique proved useful in isolating intact, individual fibers from the internodal tissues of tobacco plants. The computed flexibility co-efficient of fibers of transgenic T_1_ plants were significantly higher than those of control transformed plants (Table 1). Similarly, slenderness ratios of transgenic fibers were significantly higher than empty vector-transformed tobacco plants (Table 1). While, the lowest Runkel ratio was found in transgenic line 3 followed by 5 and 2 and the highest in vector transformed tobacco plants (Table 1).

**Table 1:** A comparative study* of physical, chemical and anatomical characteristics

**Sample**	**Slenderness ratio**	**Flexibility coefficient**	**Runkle ratio**	**Cellulose content**	**Mean number of fiber cells**
Control	17.8 ± 1.6^a^	37.6 ± 1.7^a^	1.7 ± 0.1^b^	119.0 ± 1.8^a^	22 ± 2^a^
S1	22.6 ± 1.2^b^	42.4 ± 1.1^b^	1.4 ± 0.1^a^	138.3 ± 2.6^b^	30 ± 2^b^
S2	23.1 ± 0.6^b^	44.7 ± 0.8^b^	1.2 ± 0.1^a^	131.7 ± 2.3^b^	31 ± 2^b^
S3	23.1 ± 0.9^b^	45.0 ± 2.1^b^	1.2 ± 0.1^a^	156.3 ± 2.3^c^	31 ± 2^b^
S4	22.8 ± 1.3^b^	44.0 ± 1.5^b^	1.3 ± 0.1^a^	148.3 ± 2.1^bc^	32 ± 2^b^
S5	23.6 ± 1.2^b^	44.8 ± 2.0^b^	1.2 ± 0.1^a^	165.8 ± 2.5^c^	30 ± 2^b^

*Fibers isolated from transgenic (S1-S5) and vector transformed (control) tobacco plants. Mean value followed by same superscripts within a column do not differ significantly (P ≤ 0.05) according to DMRT.

Anatomical changes concomitant with transgene expression in different transgenic lines (T_1_) revealed significant increase in the number of xylary fibers of vascular bundles compared to the empty-vector transformed tobacco stems (Table 1).

### Heterologous expression of *BbKst* in transgenic tobacco plants

**Figure 14:**
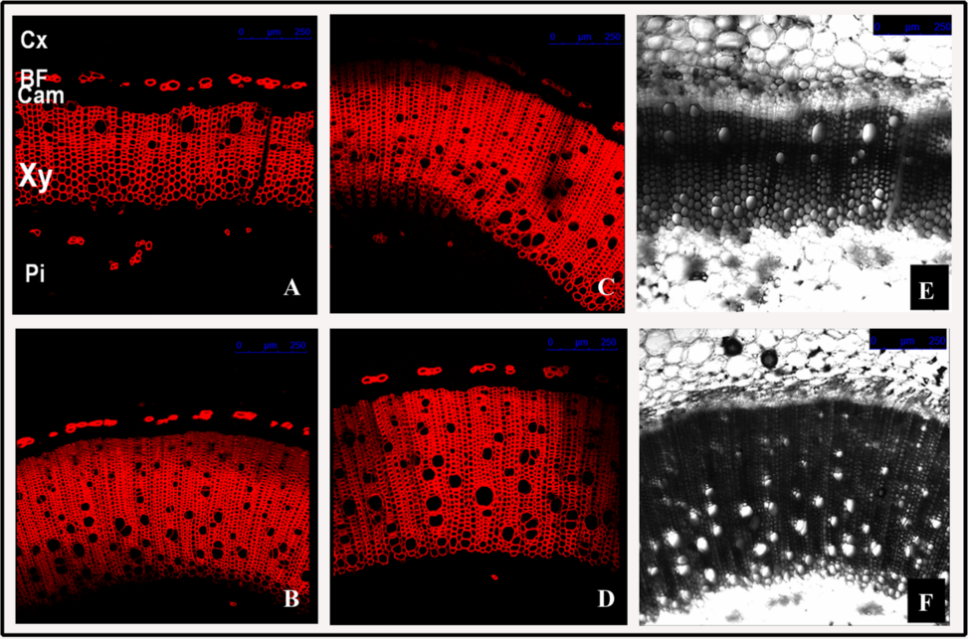
**A-F Heterologous expression of BbKst in transgenic tobacco plants T**_**1 **_**generation.** CLSM images of transverse sections of tobacco stems after 130 days of germination, labelled with Congo red; **A**, vector-transformed; **B**-**D**, transgenic lines of tobacco carrying *BbKst* gene showing enhanced xylary fibers with high cellulose content; **E**-**F** are the bright field images of **A** and **B**, respectively. Cx: Cortex; BF: Bast fiber; Cam: Cambium; Xy: Xylary fibers; Pi: Pith.

Stem cross sections of transgenic tobacco plants carrying *BbKst* were subjected to Congo-red staining as described in Methods and subsequently the stained sections were studied under CLSM. Enhanced level of cellulose depositions in the walls of xylary fibers in the stems of transgenic plants was evident. The vascular zone was more pronounced in transgenic than empty vector-transformed T_0_ plants (Additional file 6: Figure S5). Similar observations were also noted in T_1_ transgenic tobacco plants (Figure 14).

### Biochemical quantification of cellulose contents

Cellulose contents of five independent transgenic plants (S1 to S5) expressing *BbKst* gene and empty vector transformed tobacco plants were analysed as stated in method and the quantitative data are represented in Table 1. Cellulose content of these transgenic lines varied from 13.17-16.58% fresh wt. with an average of 14.82% fresh wt, whereas 11.9% cellulose content was noted in the vector-transformed tobacco plants. Overall a significant (P ≤ 0.05) 1.25 fold increase of cellulose contents in transgenic tobacco plants expressing *BbKst* gene was noted, compared to that of vector-transformed plant (Table 1).

## Discussion

In a previous study, a species specific Sequence Characterized Amplified Region (SCAR) marker was developed that discriminates *B. balcooa* from other bamboo species [1]*.* Further investigations with different natural genetic resources of *B. balcooa* yielded a second DNA marker, Balco_1128_ (GenBank ID DQ900580) that has been successfully employed to identify the elite genotypes of *B. balcooa* endowed with high cellulose contents and possessing superior fiber qualities with respect to tensile strength and durability [3]. However, the genetic linkage between Balco_1128_ and high cellulose containing trait could not be demonstrated due to the monocarpic nature and unusually long sexual cycle of bamboo [12,13].

Nevertheless, *in silico* analysis revealed the presence of an open reading frame within the marker sequence which prompted us to identify the full length sequence of *BbKst* gene. The 7375 bp long full length *BbKst* consists of 2 introns, 3 exons, 5′-3′ UTR and an upstream promoter like region. Database searches revealed the presence of key promoter-specific structural elements and many cis-acting elements in the upstream region of *BbKst* gene (Additional file 2: Table S1); among these, the RSs1 site regulates phloem specific sucrose synthesis by enhancing sucrose synthase production [14]. The deduced amino acid sequence of BbKst showed high sequence homology with other reported protein kinases from various plant taxa. The complete ORF of the protein upon BLASTP illustrated that BbKst has more than 70% homology with *Arabidopsis thaliana* (putative LRR-protein kinase, EMBL ID AAC16742.1; At1g04210), hypothetical protein of *Vitis vinifera* (GenBank ID XP_002279386.1), an ATP binding protein of *Ricinus communis* (GenBank ID XP_ 002517061.1), predicted protein of *Populus trichocarpa* (GenBank ID XP002311646.1) and hypothetical protein SORBIDRAFT_01g008430 of *Sorghum bicolor* (GenBank ID XP_002463895.1) and putative protein kinase of *Oryza sativa* var. Japonica (GenBank ID AAP13008.1) (Additional file 7: Figure S6). 5′ of the BbKst protein sequence contains six LRR motifs which may act as substrate binding sites and the 3′ end contains a serine-threonine protein kinase domain, devoid of any transmembrane domains. Pfam domain search predicted that BbKst is a receptor like cytoplasmic kinase. LRR-RLKs possess different numbers of LRR variously arranged in their extracellular domains, which are involved in protein-protein interaction and in targeting the receptors [15,16]. Plant RLCKs usually comprised of a carboxyl-terminal kinase domain but no apparent signal sequence or transmembrane domain are present. Although a large number of RLKs are present in plant genomes, function of most of these RLKs and RLCKs are not known [6,8,17]. A limited number of RLCKs are functionally characterized, for example, *Brassica* (*Brassica rapa*) M-locus protein kinase MLPK associated with self-incompatibility [18], tobacco (*Nicotiana tabacum*) NtPK1 and NtPK2 involved in pollen germination and pollen tube elongation [19], tomato (*Lycopersicon esculentum*) Pto and Pti1 in disease resistance [20], soybean (*Glycine max* L.) GmPti1 in coordinating plant defense responses [21] and *Arabidopsis* (*Arabidopsis thaliana*) CDG1 related to BR signaling [22]. There are few reports available in monocotyledonous plants, for example, ZmPti1a in maize (*Zea mays*) involved in pollen callose deposition, and ZmPti1b, in pathogen defense [23] and another reported from rice (*Oryza sativa*) OsRLCK1, involved in pollen performance during pollination [24].

There are very few evidences presuming that kinases of the RLK family have an additive role in superior fiber development. However no comprehensive study has yet been made to elucidate the biochemical nature underpinning their biological activity. In this communication we have analyzed biochemical properties of the BbKst-protein kinase, including the substrate specificity, divalent cation preference, phosphate donor specificity and potential inhibitors of BbKst-kinase activity. It was found that BbKst-kinase is a monomeric 42-kD serine/threonine protein kinase enzyme, phophorylating a broad spectrum of proteins (specifically Casein, MBP, Histone, LRRKtide, GS peptide). All kinases require divalent cations (Mg^+2^ or Mn^+2^) to coordinate the phosphate groups of the phosphate donor. BbKst, like most serine/threonine kinases, exhibited a strong preference for Mg^+2^ over Mn^+2^ in the phosphorylation reaction. BbKst lack any regulatory domains hence phosphorylation and dephophorylation appear to be main regulatory mechanisms of its activity as proposed by Kobayashi et al. [25].

Bamboo fibers are highly specialized cells with three distinct stages of differentiation [5,26]. Stage I consists of fiber cell differentiation/initiation; Stage II comprises of fiber cell elongation and at this stage secondary cell wall deposition starts; Stage III includes maturation of fiber, in which fiber cell elongation ceases and gradually additional wall layers are deposited. There is a solitary published report in the literature demonstrating genes that are differentially expressed at these three stages of fiber development [5], characterizing the cellular functions of these genes that are specifically expressed at a certain stage of fiber development. *BbKst* transcript expression was noted from Stage I of fiber development. Expression of the gene gradually increases during stage II and declined after fiber maturation.

Transformed onion epidermal cells expressing the Js1304 construct carrying *BbKst* gene coupled with *GFP* showed expression of *GFP* in the cytoplasm indicating the proper orientation of the construct and confirms that the Js1304 is functional yet in a heterologous system. Localization of GFP implied co-expression of *BbKst* in the cytoplasmic region. Further investigations by co-expressing other sub-cellular markers would reveal precise location of BbKst within the cell. Southern hybridization study revealed mostly single copy insertion in the GUS positive seedlings. Heterologous expression of *BbKst* transcripts in T_1_ lines was remarkably high in tested transgenic tobacco. The vascular region of transgenic tobacco containing *BbKst* gene was more pronounced than in the vector-transformed tobacco plants endowing additional tensile strength, which is also substantiated by their strong upright phenotypes, significantly higher flexibility coefficient, slenderness ratio, and lower Runkel ratio of the fibers. The slenderness ratio or felting power is the physical indicator for fiber durability, while the magnitude of tensile strength is usually proportional to the flexibility coefficient (26). Increase in the numbers of xylary fibers in the vascular bundles of transgenic plants was noted compared to stems of vector-transformed plants. Enhanced level of cellulose depositions in the stems of T_0_ and T_1_ transgenic plants were apparent and confirmed by quantitative analyses of cellulose contents indicating enhanced cellulose synthase activity. Such augmented cellulose deposition resulting in thicker xylem secondary cell wall formation has also been reported in the transgenic lines of hybrid poplar [27]. Plasma membrane-associated cellulose synthase (CelS) is the catalytic unit of cellulose synthesis [28] that triggers the functional cellulose-synthesis machinery during fiber development. CelS have UDP-glucose binding activity and β-1, 4-glucan (cellulose) synthesis activity. Fujii and colleagues [29] have demonstrated that sucrose synthase (SuSy) is an integral component of the cellulose synthesis machinery. In a contemporary investigation Coleman et al. [27] demonstrated close association of SuSy with cellulose synthesis during secondary wall formation. Suppression of SuSy gene expression inhibited cotton fiber cell initiation and elongation [30]. In our previous study we reported expression of SuSy gene (V1Bb154) in all the three stages of fiber development, but the highest expression was noted during fiber elongation concomitant with highest cellulose deposition [5]. However, to date there is no evidence of enhanced cellulose synthesis associated with increased number of xylem fibers. Nevertheless, cellulose-rich G (gelatinous)-layer in the walls of fiber cells generates tensile strength in the secondary xylem [31]. It is presumed from the present finding that *BbKst* has an active role in triggering SuSy production which stimulates CelS resulting in cellulose overproduction, and providing extra tensile strength of the fiber in certain genotypes of *B. balcooa* endowed with the marker/gene [3,26].

To the best of our knowledge, the results presented here are the first data describing the biochemical features of a receptor like cytoplasmic protein kinase involved in higher cellulose production and conferring superior fiber quality with high tensile strength and durability in transgenic plants. Presumably *BbKst* plays an active role in triggering sucrose synthase production which stimulates cellulose synthase resulting in cellulose overproduction, consequently providing extra physical strengths to fibers of transgenic tobacco plants endowed with *BbKst* gene.

## Conclusion

This is the first attempt to characterize a protein comprehensively at the biochemical and molecular level encoded by a gene associated with superior fiber quality in bamboo. Further comprehensive study on all interacting proteins involved in superior quality fiber formation will aid in manipulating fiber compositions required for the paper, textile or timber industries, as well as to comprehend multifunctional activities of fiber cells.

## Methods

### Plant material

Young leaf and internodes of *Bambusa balcooa* (Roxb.) were collected from natural strands of Bhadreswar (22° 49′ N 88° 21′ E), West-Bengal, India.

### RNA ligase mediated rapid amplification of cDNA ends (RLM RACE)

Total RNA was isolated from the fifth internodal tissue of bamboo following protocol adapted by Rai et al. [32]; RACE was performed using First Choice RLM RACE kit (Invitrogen, USA) following manufacturer’s instructions. Primers used in 5′ RACE were designed using the information of 5′ end sequence of the marker, Balco_1128_. Similarly 3′ end sequence information was used to design primers for 3′ RACE (Additional file 8: Table S2). Products obtained from RACE were gel purified using Qiagen gel extraction kit (Cat No 28704; QIAGEN; USA) and sequenced.

### Genomic DNA isolation and genome walking

The PCR based genome walking library was constructed by Genome Walker Universal Kit (Cat no. 638904; Clonetech, U.S.A). Briefly, high quality genomic DNA was isolated from surface sterilized tissue with 250 μl of warm CTAB extraction buffer [33], and digested with four different restriction enzymes namely, *DraI, EcoRV, PvuII, StuI* for overnight at 37°C. The digested DNA was purified, and ligated with Blunt end adaptors (25 μM, provided with the kit) for overnight at 16°C. Adaptor ligated DNA was used as template for first round genome walking PCR employing both adaptor specific (AP1, provided with the kit) and gene specific primers (Additional file 9: Table S3). The longest product was eluted from the 1% agarose gel and sequenced with BigDye Terminator Reaction Mix (Applied Biosystems, USA) using an automated DNA sequencer (ABI Prism 3100) following manufacturer’s instruction. Four rounds of walking were required to obtain the full length *BbKst* clone.

### *In-silico* sequence analysis

Both the sequences obtained from genome walking and RACE were aligned by NCBI blast alignment using Bioedit software. Positions of promoter sequence, transcription start site, introns and exons were determined by NCBI- blast and by multiple alignments with ClustalW (http://www.ebi.ac.uk/Tools/msa/clustalw2/). The *in silico* translated amino acid sequence derived from the *BbKst* gene was further analyzed for conserved domains and motifs using ProSite and Inter-Proscan.

### Bacterial expression of BbKst- kinase cDNA

#### Expression cassette construction

The kinase domain of BbKst (from 2233 to 3342 bp of cDNA as shown in Figure 1C) was cloned in *EcoRI* and *SalI* sites of pET28a(+) vector. Subsequently transferred in BL21 cells, plasmid DNA obtained from a positive clone was sequenced with gene specific primers for sequence conformation. BbKst-kinase-His fused protein was induced with IPTG and purified using Ni- NTA nickel column (Cat no. 31014; Qiagen) following manufacturer’s instruction.

### *In vitro* kinase assays

Substrate phosphorylation experiments were carried out employing 10 μg of BbKst-kinase in 50 μl of reaction mixture (25 mM Tris-HCl; pH 7.5, 1 mM DTT; 5 mM MgCl_2_ and 10 μM ATP) in presence of 10 μM [γ ^-32^P] ATP (3000 cpm pmol^-1^) at 30ºC with 250 mM of different substrates viz. casein, myelin basic protein (MBP), histone (H1), LRRKtide and GS peptide. Phosphorylated products were analysed by SDS-PAGE and autoradiographed. Activity of BbKst-kinase was determined by plotting its specific activities with varying concentrations of casein as substrate. Steady-state kinetic parameters were evaluated using Michaelis-Menten kinetics. The *V*_*max*_ and *K*_m_ value of BbKst-kinase using casein as a substrate following Eadie-Hofster plots of V_0_ versus V_0_/[S]. The turnover number was calculated from K_cat_ at the optimal substrate concentration per active site and the average time required to hydrolyze one molecule of casein was determined.

### Effect of protein kinase inhibitors of BbKst-kinase activity

Several potent inhibitors of protein kinases like heparin, H-89, quercetin and staurosporine were tested on BbKst-kinase following recommended methods of Davies et al. [34].

### Phospho-amino acid analysis

The purified BbKst protein was labeled *in vitro* with [γ-^32^P] ATP and autophosphorylated protein electroblotted onto a polyvinylidine difluoride membrane. Radioactive band of interest was excised and hydrolyzed in 6 N HCl for 2 hr at 110°C [35]. The hydrolysate was concentrated and spotted on TLC plate and analysed along with Phosphoamino acid standards such as phospho- Ser, phospho-Thr, and phospho-Tyr (25 nM each; Sigma). TLC plates was then exposed to X-ray film and subsequently developed, phosphoamino acids standards were visualized by ninhydrin spraying over the TLC plate.

### RNA slot blot hybridization

Different intermodal tissues (viz. I2, I5, I8, I10, I12, I15) were collected from three independent clumps of *B. balcooa* separately and processed for extracting total RNA as described earlier (35). Twenty μg RNA of each sample were loaded in formaldehyde denatured agarose gel and electrophoresed at a constant voltage of 50 V. The RNA was transferred to Hybond-N + membrane (Amersham-Pharmacia Biotech) with Bio-Rad Trans-Blot SD Semi-Dry Electrophoretic Transfer Cell at a constant current of 400 mA for 45 min. 1265 bp long cDNA fragment was radiolabelled with [α-^32^P]dCTP using Prime-a-gene labeling system kit (Promega, USA) as per the manufacturer’s instructions. The membrane was incubated with the ^32^P labeled gene specific probe at 65°C overnight in hybridization buffer (Sigma). After hybridization the membrane was washed twice with 2× SSC containing 0.1% SDS for 30 min each at 65°C followed by washing for 30 min (2 times) in 1× SSC and 0.5× SSC containing 0.1% SDS at 65°C. The membrane was exposed to X-Ray films for 24 hr. with intensifying screen and films were developed. The same procedure was followed for GAPDH as control. All experiments were repeated three times.

### qPCR analyses of *BbKst* transcripts in bamboo

cDNA was synthesized from the total RNA of leaf, stem, different internodal tissues (2^nd^, 5^th^, 8^th^, 10^th^, 12^th^ and 15^th^ from the tip) and rhizome of *B. balcooa* using First strand cDNA synthesis Kit (Cat # K1622. Fermentas). qPCR reactions were performed in presence of gene specific primers using a LightCycler PCR System qPCR instrument (MJ Research, Bio-Rad; Model CFD-3220). Fold differences in the *BbKst* transcript level were quantified using the 2^ΔΔC^_T_ method [36,37], considering fold difference of GAPDH as 1.0.

### Construction of plant expression vector and *Agrobacteria* mediated transformation

The full length 3339 bp cDNA of BbKst was inserted between *Spe1* and *Nco1* restriction sites in a sense orientation in pCambia 1304. Positive construct was designated as Js1304 (Figure 9). *Agrobacterium tumefaciens* strain C58C1 was transformed with Js1304 by heat shock method. Js1304 integrated-*Agrobacteria* mediated transformation of tobacco was performed following the protocol of Kumar et al. [38]. Ten independent transgenic tobacco lines were generated with the Js1304 construct and maintained in the green house until seeds were mature. Seeds were harvested and germinated in presence of 30 ug^-ml^ hygromycin and segregation ratio was analyzed.

### Transient expression of the *BbKst*

Gold-spermidine mixture (25 mg of 1 μm gold particles + 100 μl spermidine) was added to 25 μg of Js1304 plasmid DNA and mixed prior to the addition of 100 μl of 1 M CaCl_2_. The onion peels were placed on the wet filter paper and kept in a Petridish. The gold particles were fired at 200 psi from a 3 cm distance of a Particle Gun (Helios Gene Gun, Bio-Rad make). Each onion peel was put in the Petri dish keeping the epidermal side in contact with the Murashige and Skoog’s (MS) medium [39]. Petridishes were sealed with parafilm and kept at 23˚C for 24 hr. Each onion piece was taken on a glass slide and GFP fluorescence was visualized and image captured in confocal laser scanning microscope (CLSM) using LAS AF (Leica Application Suite Advanced Fluorescence, 1.8.1) software with a confocal pinhole set at “Airy” 5.50 and zoomed to a factor of 1.4× for improved 8- bit resolution. Materials were excited at 488 nm wave length and emission was at 518 nm.

### GUS histochemical assay

Histochemical GUS assay was performed following the method of Jefferson [40] in order to monitor the transient gene expression in 21 days old T_1_ plants. Tissues were incubated in 1 mM chromogenic substrate XGlcA (5-bromo-4-chloro-3-indolyl-β-D-glucuronic acid) containing GUS substrate solution for overnight at 37°C. Then tissues were fixed in the fixative for at least 4 hrs followed by treatment with 50% and 100% ethyl alcohol for complete decolorization. Finally tissues were transferred to GUS fixative solution and examined under light microscope and photographed.

### Southern hybridization with partial kinase domain of *BbKst* as probe

Total genomic DNA from young leaves of GUS-positive transgenic plants and vector-transformed plants was isolated following the protocol mentioned above. For Southern analysis, approximately 10 μg of genomic DNA was digested with 100 units (about 10 U/μl) of two enzymes Spe1 and Nco1, electrophoresed in a 0.8% agarose gel and subsequently transferred to a Hybond-N + membrane (Amersham-Pharmacia Biotech). DNA fragment of 1265 bp was amplified with JNWF1 (5′-CTAAAAGGCACCCTGATATGGACAGCATC-3′; Additional file 9: Table S3); and JF3R (5′-GCAAGAAGCATGTACCTATTGAC-3′) primers and then eluted from the gel, 25-50 ng of the eluted DNA was used as a probe after labelling with [α-^32^P]-dCTP using Prime-a-gene labeling kit (Promega, USA) as per manufacturer’s instructions. Hybridization was carried out overnight at 65°C. After hybridization the membrane was washed twice with 2× SSC buffer containing 0.1% SDS for 30 min each at 65°C followed by washing for 30 min (2 times) in 1× SSC and 0.5× SSC buffers containing 0.1% SDS at 65°C. The membrane was covered with the saran-wrap, exposed to X-ray film (Kodak) with intensifying screen and kept at -80 ºC for 48 hr.

### Expression analysis of *BbKst* in transgenic tobacco plant

Expression analyses of *BbKst* in transgenic tobacco plants and empty vector transformed plants were carried out in Light-Cycler PCR System qPCR instrument (MJ Research, Bio-Rad; Model CFD-3220). Primers used for qPCR were 5′-CCTCGGTGTTCCTTCCCTTCTGTC-3′ and 5′-GTCAATAGGTACATGCTTCTT GC-3′. The reaction conditions were as follows: initial denaturation at 95°C for 5 min, followed by 40 cycles of denaturation at 94°C for 45 sec, annealing at 56°C for 30 sec, and extension at 72°C for 1 min, final elongation step at 72°C for 10 min. Melting curve was analysed by continuous monitoring of fluorescence between 60°C and 95°C with 0.5°C increment after every 30 sec. Tobacco 18S rRNA house keeping gene was used as internal control. Fold differences in the *BbKst* transcript level were quantified using the 2^-ΔΔC^_T_ method [36,37], considering fold difference of 18S rRNA as 1.0.

### Measurement of stem thickness of transgenic tobacco plants expressing *BbKst* gene

The stem thickness (diameter) of 5 month old transgenic tobacco plants and empty vector-transformed control tobacco plants grown in green house conditions (28°C and 78% humidity) were measured with slide calipers. Measurements of 7^th^ to 20^th^ internodes from tip of these plants were taken. Three independent measurements from each internode were noted and the mean value was scored. Width of the xylem zone (xylem vessels and xylary fibers) of independent transgenic and control plants were measured under the microscope from at least 5 samples and the mean value was tabulated. Width of xylem zone (containing xylary fibers and vessels) of five independent transgenic lines was compared with respective stem width. Similar comparison was also made for one vector-transformed tobacco plant.

### Physical characteristics of fiber cells

Small slivers were prepared from 19^th^ internode (from the tip) by macerating the tissue with 67% nitric acid, boiled in a water bath for 10 min and subsequently washed thoroughly in distilled water following the protocol of Bhattacharya et al. [26]. Measurements of fiber diameter, cell wall thickness and lumen diameter (i.e. fiber diameter - cell wall thickness) of 25 intact fiber cells isolated from five T_1_ transgenic plants were taken. The following equations were used to derive the physical traits: slenderness ratio = fiber length/fiber diameter, flexibility coefficient = (fiber lumen diameter/ fiber diameter) X 100 and Runkel ratio = (2 X fiber cell wall thickness)/lumen diameter [26]. Duncan’s Multiple Range Test (DMRT) was performed to analyze the results proximity with each other, the test was made in SPSS statistical software.

### Histological analysis of cellulose contents of fiber cells

Transverse hand sections were taken from the 18^th^ internodes (from tip of the stem) of vector- transformed and transgenic tobacco plants. Sections were stained with 0.2% aqueous solution of Congo Red (CR; Sigma, C6767) overnight in the dark following the standardized protocol prescribed by Bhattacharya et al. [26]. Briefly, the stained sections were washed with deionized water, and images were acquired using CLSM with a confocal pinhole set at ‘Airy’ 1.0. A 30% Argon laser with Acousto-Optical Tunable Filter (AOTF) for 514 nm (20 mW) was used for exciting CR-labeled samples, and red fluorescence emissions were acquired between 560 and 800 nm with the PMT detector gain set at 1100 V. Fluorescence was recorded after passage through a triple dichroic filter 458/514/594. Images were captured using a HCX PL APO objective (63X/NA 1.40) under the above stated conditions.

### Estimation of cellulose contents in transgenic plants

The α-cellulose content was estimated following the colorimetric method based on anthrone reagent [26,41]. Approximately 1.0 g of 3^rd^ internodal tissues from base were crushed, mixed with 3 ml of nitric acid and acetic acid solution (1:8 v/v) and refluxed in boiling water for 30 min. Lignin, hemicellulose and xylosans were removed through successive three washing followed by centrifugation at 10000 rpm for 10 min. The resultant pellet was dissolved in 67% sulphuric acid (v/v), mixed with chilled anthrone reagent (HiMedia Laboratories, India), and incubated for 20 min in a boiling water bath. Cellulose estimation was made spectrophotometrically at 620 nm (Beckman-Coulter, DU-520). The α-cellulose content was estimated from the standard curve prepared using different known concentrations of cellulose (Merck, Germany) and was expressed as the gram percentage of fiber fresh weight.

## Competing interests

We hereby declare that we have no financial or non-financial competing interest.

## Authors’ contributions

AP designed and coordinated the entire study, participated in developing SCAR markers and writing the manuscript. JSG did almost all experimental and bioinformatics work under the guidance of AP and partially drafted the manuscript. SC participated in the design of all the biochemical experiments and guided JSG also participated in editing the manuscript. ND supervised the entire transgenic work and helped in manuscript preparation. All authors read and approved the final manuscript.

## Authors’ information

JSG is now working as a post-doc at the Department of Agronomy, Iowa State University, USA. ND is a scientist at the Institute of Life Sciences, Bhubaneswar, India and is a member of the gene function and regulation group, mainly contributes towards development of novel promoters with enhanced activity. SC is an Assistant Professor at the Bose Institute, India and is mainly working on the epigenetic regulations during plant response to environmental or development cues. AP is a Senior Professor in the Department of Plant Biology, Bose Institute, India and is involved mainly with molecular mechanism of plant pathogen interaction also involved in the development and use of molecular markers. She is interested in understanding the molecular basis of fiber development in tree bamboo and contributed in species and genotype specific DNA marker development in bamboo species.

## Supplementary Material

Additional file 1: Figure S1 Ai. RLM-RACE: 5′ represents the nested 5′ RACE product; 3′ represents the 3′ RACE product. Aii. Genome walking in 5′ (left panel) and 3′ (right panel) direction. Click here for file

Additional file 2: Table S1 Significant cis-elements (apart from structural elements) found in the upstream region of *BbKst*. Click here for file

Additional file 3: Figure S2 Phenotypes of transgenic tobacco plants of T1 generations. Click here for file

Additional file 4: Figure S3 A-D GUS gene expression in *BbKst* transgenic plants. Click here for file

Additional file 5: Figure S4 Single copy insertion of *BbKst* in tobacco transgenics. Click here for file

Additional file 6: Figure S5 A-F Heterologous expression of *BbKst* in transgenic tobacco plants of T0 generation. Click here for file

Additional file 7: Figure S6 Sequence homology of BbKst with other plant kinase proteins. Click here for file

Additional file 8: Table S2 Primers used for RLM-RACE. Click here for file

Additional file 9: Table S3 Primers used for genome walking. Click here for file
