# Supplementary material for: Functional characterization of a serine-threonine protein kinase from Bambusa balcooa that implicates in cellulose overproduction and superior quality fiber formation
Source: BMC Plant Biol. 2013 Sep 10;13:128. doi: 10.1186/1471-2229-13-128 (PMC3847131; doi:10.1186/1471-2229-13-128)
Supplement: Additional file 1: Figure S1 — Ai. RLM-RACE: 5′ represents the nested 5′ RACE product; 3′ represents the 3′ RACE product. Aii. Genome walking in 5′ (left panel) and 3′ (right panel) direction. [file 1471-2229-13-128-S1.doc]

**Additional file** 1 Figure S1**:** **Ai**. RLM-RACE: 5' represents the nested 5' RACE product; 3’ represents the 3' RACE product. M is the molecular weight marker (1.5kb + 100bp ladder). **Aii.** Genome walking in 5' (left panel) and 3' (right panel) directions. Secondary PCR products from PvuII, EcoRV and StuI library marked with 1, 2 and 3, respectively. M is marker of 100 bp.
