# Supplementary material for: Functional characterization of a serine-threonine protein kinase from Bambusa balcooa that implicates in cellulose overproduction and superior quality fiber formation
Source: BMC Plant Biol. 2013 Sep 10;13:128. doi: 10.1186/1471-2229-13-128 (PMC3847131; doi:10.1186/1471-2229-13-128)
Supplement: Additional file 2: Table S1 — Significant cis-elements (apart from structural elements) found in the upstream region of BbKst. [file 1471-2229-13-128-S2.doc]

***Additional file 2*** *Table S1. Significant cis-elements (apart from structural elements) found in the upstream region of BbKst.*

| **Name of the Element** | **Motif id** | **Motif sequence** | **Matched species** | **Function** | **Reference** |
| --- | --- | --- | --- | --- | --- |
| ABRE-like sequence | S000414 | ACGTG | *Arabidopsis* | ABRE-like sequence required for etiolation-induced expression of erd1 (early responsive to dehydration) | Nakashima et al., 2009 [1]. |
| amylase box | S000021 | TATCCAT | Rice, Barley | Conserved sequence found in the alpha amylase gene | Huang et al., 1990 [2]. |
| G-box | S000345 | CACGTG | *Arabidopsis* | Induced and repressed phyA-responsive promoters | Hudson and Quail, 2003 [3]. |
| Myc recognition element | S000407 | CATGTG | *Arabidopsis* | Dehydration-inducible expression of the *ERD1* gene | Phan Tran et al, 2004 [4]. |
| RSs1 | S000128 | AATCCAA | Rice | Phloem specific, essential for sucrose synthesis | Saha et al., 2007 [5] |
| ABRE related sequence | S000671 | CCACGTCA | Rice | ABRE related sequence | Hobo et al., 1999 [6]. |

**References:**

1. Nakashima K, Yusuke I, Yamaguchi-Shinozaki K: **Transcriptional regulatory networks in response to abiotic stresses in Arabidopsis and grasses**. Plant Physiol 2009, **149 (1):** 88-95 [URL: <http://www.plantphysiol.org/content/149/1/88.full>].
2. Huang N, Sutliff TD, Litts JC, Rodriguez RL: **Classification and characterization of the rice alpha-amylase.** Plant Mol Biol 1990, **14:** 655-668. [URL: <http://www.ncbi.nlm.nih.gov/> pubmed/2102847]
3. Hudson ME, Quail PH: **Identification of promoter motifs involved in the network of phytochrome A-regulated gene expression by combined analysis of genomic sequence and microarray data.** Plant Physiol 2003, **133:**1605-1616. [URL: [http://www.plantphysiol](http://www.plantphysiol/). org/content/133/4/1605.full].
4. Phan Tran LS, Nakashima K, Sakuma Y, Simpson SD, Fujita Y, Maruyama K, Fujita M, Seki M, Shinozaki K, Yamaguchi-Shinozaki K: **Isolation and functional analysis of Arabidopsis stress-inducible NAC transcription factors that bind to a droughtresponsive *cis*-element in th*e* early responsive to dehydration stress 1 promoter** . Plant Cell 2004, **16:** 2481-2498. [URL: <http://www.plantcell.org/content/16/9/2481.full>].
5. Saha P, Chakraborti D, Sarkar A, Dutta I, Basu D, Das S: **Characterization of vascular-specific *RSs1* and *rolC* promoter for their utilization in engineering plants to develop resistance against hemipteran insect pests.** Planta 2007, **226:** 429-442. [ URL: http://www.springerlink.com/content/ek6x0637866713q1/].

Hobo T, Asada M, Kowyama Y, Hattori T: **ACGT-containing abscisic acid response element (ABRE) and coupling element 3 (CE3) are functionally equivalent.** Plant J 1999, **19(6):** 679-689. [ URL: <http://onlinelibrary.wiley.com/doi/10.1046/j.1365-> 313x.1999.00565.x/pdf].
