# Supplementary material for: Functional characterization of a serine-threonine protein kinase from Bambusa balcooa that implicates in cellulose overproduction and superior quality fiber formation
Source: BMC Plant Biol. 2013 Sep 10;13:128. doi: 10.1186/1471-2229-13-128 (PMC3847131; doi:10.1186/1471-2229-13-128)
Supplement: Additional file 3: Figure S2 — Phenotypes of transgenic tobacco plants of T1 generations. [file 1471-2229-13-128-S3.doc]

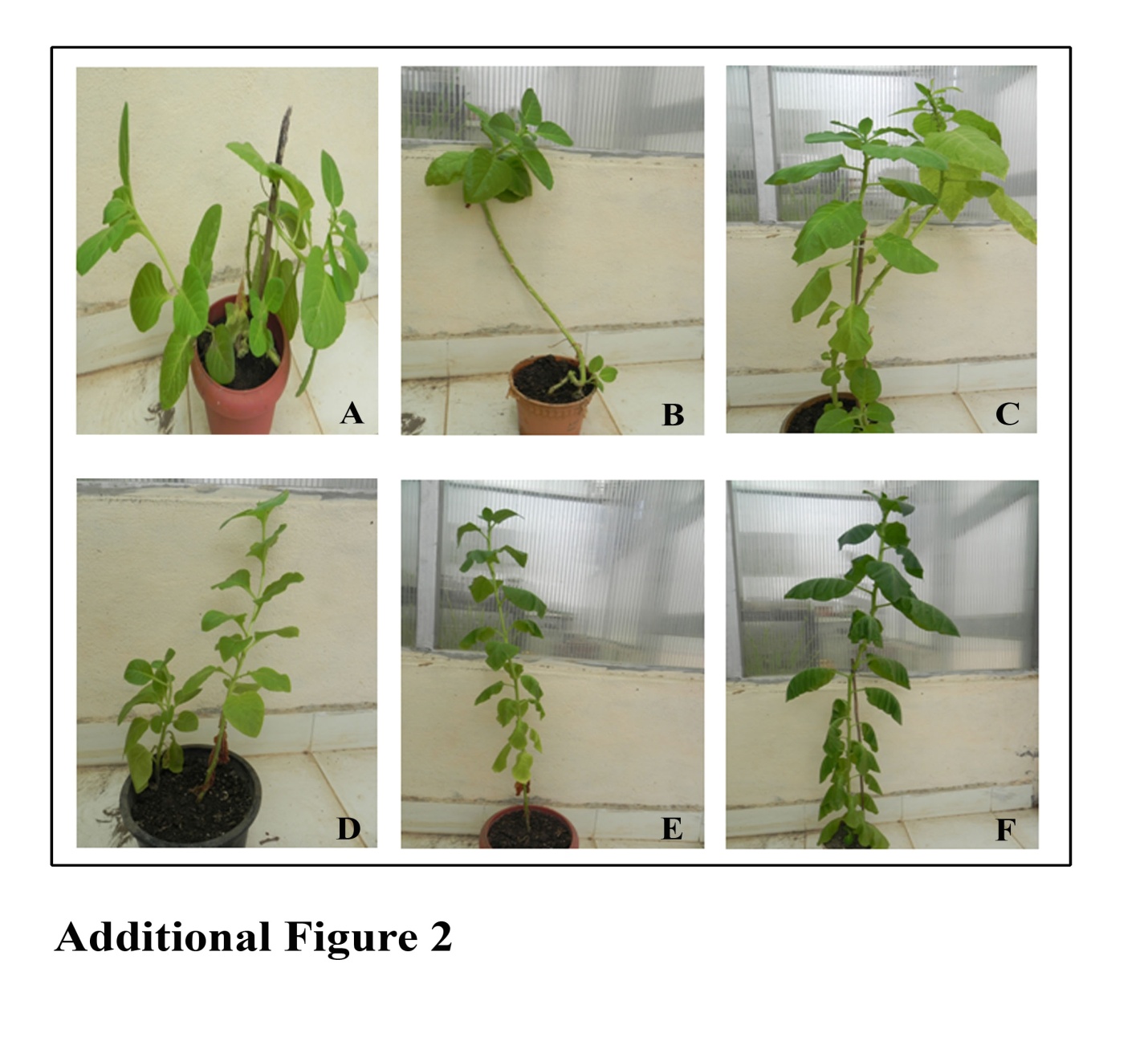
**Additional file 3** Figure S2**: Phenotypes of transgenic tobacco plants of T_1_ generations.** Upper panel (A- C) showing the vector-transformed tobacco plants and lower panel (D- F) showing *BbKst* transgenic tobacco plants.
