# Supplementary material for: Functional characterization of a serine-threonine protein kinase from Bambusa balcooa that implicates in cellulose overproduction and superior quality fiber formation
Source: BMC Plant Biol. 2013 Sep 10;13:128. doi: 10.1186/1471-2229-13-128 (PMC3847131; doi:10.1186/1471-2229-13-128)
Supplement: Additional file 4: Figure S3 A-D — GUS gene expression in BbKst transgenic plants. [file 1471-2229-13-128-S4.doc]

**Additional file 4** Figure S3**:** **GUS gene expression in *BbKst* transgenic plants.** A-C: GUS gene expression of independent events in T_1_ generation, transgenic seedlings showing blue coloration suggesting integration of *BbKst* gene. D: Vector-transformed tobacco seedling as negative control.
