# Supplementary material for: Functional characterization of a serine-threonine protein kinase from Bambusa balcooa that implicates in cellulose overproduction and superior quality fiber formation
Source: BMC Plant Biol. 2013 Sep 10;13:128. doi: 10.1186/1471-2229-13-128 (PMC3847131; doi:10.1186/1471-2229-13-128)
Supplement: Additional file 5: Figure S4 — Single copy insertion of BbKst in tobacco transgenics. [file 1471-2229-13-128-S5.doc]

**Additional file 5** Figure S4**: Single copy insertion of *BbKst* in tobacco transgenics.** Southern blot analyses of genomic DNA from four transgenic T_1_ plants (S1 to S4) using 1265 bp of kinase domain as probe, showing prominent positive signal in all cases. Vector-transformed plant used as negative control (VC).
