# Supplementary material for: Functional characterization of a serine-threonine protein kinase from Bambusa balcooa that implicates in cellulose overproduction and superior quality fiber formation
Source: BMC Plant Biol. 2013 Sep 10;13:128. doi: 10.1186/1471-2229-13-128 (PMC3847131; doi:10.1186/1471-2229-13-128)
Supplement: Additional file 6: Figure S5 A-F — Heterologous expression of BbKst in transgenic tobacco plants of T0 generation. [file 1471-2229-13-128-S6.doc]

**Additional file 6** Figure S5**: Heterologous expression of *BbKst* in T_0_ transgenic tobacco plants**. CLSM images of transverse sections of tobacco stems after 120 days of germination labelled with Congo red; **A**, vector-transformed **B-D**, transgenic lines of tobacco carrying *BbKst* gene showing enhanced xylary fibers with high cellulose content; **E-F** are the bright field images of **A** and **B**, respectively. Cx: Cortex; BF: Bast fiber; Cam: Cambium; Xy: Xylary fibers; Pi: Pith.
