# Supplementary material for: Functional characterization of a serine-threonine protein kinase from Bambusa balcooa that implicates in cellulose overproduction and superior quality fiber formation
Source: BMC Plant Biol. 2013 Sep 10;13:128. doi: 10.1186/1471-2229-13-128 (PMC3847131; doi:10.1186/1471-2229-13-128)
Supplement: Additional file 7: Figure S6 — Sequence homology of BbKst with other plant kinase proteins. [file 1471-2229-13-128-S7.doc]

**Additional file 7** Figure S6**: Sequence homology of BbKst with other plant kinase proteins.** BLASTP result show high sequence homology of the deduced amino acid sequence of kinase domain of BbKst with protein kinases of *Arabidopsis thaliana* (AAC16742.1) and *Vitis vinifera* (XP_002279386.1); an ATP binding protein of *Ricinus communis* (XP_002517061.1), predicted protein kinase of *Populus trichocarpa* (XP_002311646.1) and hypothetical protein of *Sorghum bicolor* SORBIDRAFT_01g008430 (XP_002463895.1), putative protein kinase of *Oryza sativa* var. Japonica (AAP13008.1). Conserved aminoacids are represented in red colour (consensus value >90%) and less conserved including conservative replacements are shown in blue (consensus value >50%).
