# Supplementary material for: Functional characterization of a serine-threonine protein kinase from Bambusa balcooa that implicates in cellulose overproduction and superior quality fiber formation
Source: BMC Plant Biol. 2013 Sep 10;13:128. doi: 10.1186/1471-2229-13-128 (PMC3847131; doi:10.1186/1471-2229-13-128)
Supplement: Additional file 8: Table S2 — Primers used for RLM-RACE. [file 1471-2229-13-128-S8.doc]

**Additional file 8** Table S2: Primers used for RLM-RACE.

| **Primer Code** | **Primer Sequence (5’-3’)** |
| --- | --- |
| JR1 | CGCAGATCCGACGCCACCGACATTGA |
| JNR1 | GAGCTCCCCCTTCTCCTTCCATCCTG |
| JR2 | GCAAGCACCAACAGTTCAGCAGAACCT |
| JNR2 | CTTGAAAGCCTTGACGTATCAAACAAC |
| JR3 | GCAGATCGATACTCTTTCTGGGCAC |
| JNR3 | TACTTGTGCGATCGAGCAGACCCTCCAAC |
| JR4 | GTATTGCAGTCTATAATTCTAATGGAGTA |
| JNR4 | CATACATGCTGTATAGCTCACCTTGGCAC |
| JR5 | AGTAGATATCTGGTCATTTGGATGTT |
| JNR5 | GATCACATCTGGTGCTCATGCAGAGAA |
| JR6 | CATATGTGACAGCAATGAAGCATCTAAG |
| JNR6 | ACAGCTTCTAGTTCAGTCTACTACTGCAC |
