# Supplementary material for: Functional characterization of a serine-threonine protein kinase from Bambusa balcooa that implicates in cellulose overproduction and superior quality fiber formation
Source: BMC Plant Biol. 2013 Sep 10;13:128. doi: 10.1186/1471-2229-13-128 (PMC3847131; doi:10.1186/1471-2229-13-128)
Supplement: Additional file 9: Table S3 — Primers used for genome walking. [file 1471-2229-13-128-S9.doc]

**Additional file 9** Table S3: Primers used for genome walking.

| **Primer Code** | **Primer Sequence (5’-3’)** |
| --- | --- |
| JWF1 | CACTGATCCTGCTTGTTTGAGCAGCTGTG |
| JNWF1 | CTAAAAGGCACCCTGATATGGACAGCATC |
| JWF2 | AGGAAAGCATATCATAACTTGGCACCAC |
| JWNF2 | CAACAAGAACGTTTACCTCTGTGAGC |
| JWF3 | GCACACCAGTAGTCAAACTCTCTGA |
| JWNF3 | GAGATGCTTACGCTTCGGATACGCAC |
| JWF4 | CTTTCATGCTCACACCCAGAAACATC |
| JWNF4 | CAAGTGCCTCACCAAGCTCACAATC |
| JWF5 | GACGGCATCCAGGACGACGACATCCAGCGCA |
| JWNF5 | GATCTGGACTCCAGCCGATCCCTGCTCTG |
| JWF6 | CTGTTCCTTTTGAATGAACGACATCTTC |
| JWNF6 | GCCACTTTTGAGGGAAGGACTAGCAATG |
| JWF7 | GCCACTTTTGAGGGAAGGACTAGCAATGAC |
| JWNF7 | AGACGGTATCAAATAATAATGGCAATAATG |
